# The effect of lysophosphatidic acid on myometrial contractility and the mRNA transcription of its receptors in the myometrium at different stages of endometrosis in mares

**DOI:** 10.1186/s12917-024-04384-2

**Published:** 2024-12-19

**Authors:** Katarzyna Karolina Piotrowska-Tomala, Anna Szóstek-Mioduchowska, Agnieszka Walentyna Jonczyk, Ewa Monika Drzewiecka, Michał Hubert Wrobel, Takuo Hojo, Graca Ferreira-Dias, Dariusz Jan Skarzynski

**Affiliations:** 1 Institute of Animal Reproduction and Food Research, Polish Academy of Sciences, 10-748, Olsztyn, Poland; 2 Kyushu Okinawa Agricultural Research Center, NARO, 2421, Suya, Koshi, Kumamoto 861-1192 Japan; 3 Faculty of Veterinary Medicine, C.I.I.S.A, University of Lisbon, Lisbon, Portugal; 4 AL4AnimalS-Associate Laboratory for Animal and Veterinary Sciences, Lisbon, Portugal

**Keywords:** Lysophosphatidic acid, Lysophosphatidic acid receptor, Myometrium, Force of contraction, Uterus, Mare, Endometrosis

## Abstract

**Background:**

Endometrosis (chronic degenerative endometritis) results in morphological changes in the equine endometrium and impairs its secretory function. However, the effect of this condition on the myometrium remains unclear. Lysophosphatidic acid (LPA) may affect female reproductive function and embryo transport by influencing uterine contractility through its receptors (LPARs). The objective of this study was to determine myometrial *LPAR1–6* mRNA transcription, and the effects of LPA on myometrial contractions in mares with endometrosis during the mid-luteal and follicular phases of the estrous cycle.

**Results:**

A reduction in myometrial *LPAR1* mRNA transcription was observed in mares with endometrosis during the mid-luteal phase, in comparison to those with category I endometria (*P* < 0.05). While, upregulation of myometrial *LPAR3* or *LPAR6* mRNA transcription was observed in mares with category III or IIB endometria; respectively (*P* < 0.05). An increase in myometrial *LPAR1*, *LPAR3* and *LPAR5* mRNA transcription was observed during the follicular phase in mares with category IIA endometrium in comparison to their expression in category I endometrium (*P* < 0.05). During endometrosis progression LPA reduced the force of myometrial contractions in both phases of the estrous cycle (*P* < 0.05). However, in mares with category IIA endometrium during the follicular phase, LPA was found to increase the force of contraction of myometrial strips in comparison to mares with category I endometrium (*P* < 0.01).

**Conclusion:**

In the course of endometrosis in mares, a disruption in the myometrial mRNA transcription of *LPARs* has been observed. This is the first study to examine the impact of LPA on myometrial contractility at diffrent stage of endometrosis. However, it is essential to consider that multiple factors may contribute to this process. Alternations in contractile activity and changes in myometrial *LPARs* mRNA transcription may indicate impaired LPA-signaling mechanisms in equine myometrium during endometrosis.

## Background

Equine endometrosis is a chronic degenerative condition characterized by the formation of fibrosis in the endometrial stroma and around the endometrial glands. In addition, pathological changes in the endometrial glands, including cystic dilation, epithelial atrophy or hypertrophy, and dilation of lymphatic vessels, are observed in equine endometrium [1–4]. Endometrosis disrupts the structure of the endometrial tissue and affects the secretory function of endometrial cells [3, 5, 6]. This leads to changes in the uterine microenvironment and disruption of the processes that occur during early pregnancy [3, 7]. Endometrosis is a leading cause of subfertility and infertility in mares, resulting in significant economic losses to the horse industry.

Numerous studies have investigated the pathogenesis of endometrosis and the secretory function of the endometrium during this condition [8–10]. However, the potential impact of endometrosis on myometrial function remains to be evaluated. Hanada et al. [11] have reported that endometrosis is associated with structural changes in the myometrium. Mares with endometrosis have atrophy of the uterine smooth muscle, fatty degeneration of atrophic myocytes, and hyperplasia of collagen fibers among the smooth muscle of the myometrium [11]. According to LeBlanc et al. [12], endometrosis may be associated with impaired lymphatic circulation and fluid removal from the uterus. In addition, vascular degeneration has been demonstrated in myometrial vessels and in large arteries and veins between the circular and longitudinal myometrial layers [12]. Troedsson and Liu [13] and Troedsson et al. [14] reported that equine uteri with chronic infection have reduced uterine smooth muscle function due to poor myoelectric tone.

To the best of our knowledge, there is a lack of studies in mares investigating the myometrial receptivity to factors that may regulate its contractile activity. The present pilot study is focused on one such factor, namely lysophosphatidic acid (LPA). This extracellular lipid activates specific cell surface receptors that are members of the transmembrane G-protein-coupled receptors (GPCRs) superfamily, including LPA receptors (LPAR) 1–3, or the purinergic receptor family, including LPAR4–LPAR6 [15–20]. Lysophosphatidic acid performs a number of different biological functions, including the promotion of cell growth, differentiation, movement, survival, and cytoskeleton morphological changes [21]. Previous studies have confirmed that LPA plays a crucial role in reproductive processes, embryonic development, implantation, and pregnancy establishment in range of species including rats [22, 23], mice [24], pigs[25], cows[26–29], and sheep [30, 31]. It has been demonstrated that LPA affects uterine smooth muscle contractions during the estrous cycle, early pregnancy, and labor in humans [32], rats [22, 33], mice [24], and pigs [34]. The proper function of the uterus depends on these contractions for the physical clearance [35, 36]. Therefore, equine myometrial contractions are crucial for gamete transport [37, 38] and the mobility of the embryo within the uterine lumen [37]. Insufficient or hyperactive contractions have been associated with infertility and implantation failure in mares [39]. Furthermore, they can result in the development of uterine infection [14]. However, it remains uncertain whether LPA can regulate myometrial contractions in mares. Recently, Szóstek-Mioduchowska et al. [40] demonstrated alterations in the concentration of LPA in the endometrium, as well as in the expression and protein abundance of its receptors at different stages of endometrosis. To the best of our knowledge, the transcription of mRNA *LPARs* in the myometrium during different stages of equine endometrosis has not yet been described. Additionally, the impact of LPA on myometrial contractility in mares during endometrosis remains unknown. This study hypothesizes that (1) *LPAR* mRNA transcription is disrupted in equine myometrium during endometrosis, and (2) the effect of LPA on myometrial contractility depends on the stage of endometrosis in mares. Therefore, the objective of this study was to establish the transcription of mRNA *LPAR1-6* in the equine myometrium and to investigate the impact of LPA on myometrial contractions in different Kenney and Doig's mare endometrium categories during the mid-luteal and follicular phases of the estrous cycle.

## Methods

### Material collection

Post-mortem myometrial tissues were collected from 83 mares (Polish Coldblood horse) with ovarian cyclicity, weighing 500 ± 100 kg and ranging from 2 to 18 years of age. The mares were confirmed to be clinically healthy by an official government Veterinary inspector and by referral to historical health records for each animal. The animals were slaughtered as part of routine protocols to obtain meat at a local abattoir (Rawicz, Poland). The mares were slaughtered in accordance with European legislation (EFSA, AHAW/04–027) to eliminate pain and suffering. The Local Ethics Committee for Experiments on Animals in Olsztyn, Poland (Agreements No. 51/2011) approved all material collection procedures. The uteri were collected within 5 min of the mare's death. Prior to euthanasia, peripheral blood samples were collected into heparinised tubes (Monovettes-Sarstedt, Nümbrecht, Germany) for later progesterone (P_4_) analysis.

Myometrial samples were linked to the endometrium that was previously assigned according to Kenney Doig endometrial histophatological grading [2]. The endometrium was collected from all uteri from which myometrium was obtained. The endometrium was washed with cold sterile RNAse-free saline solution and placed into 4% buffered paraformaldehyde (POCH, Gliwice, Poland, #432,173,111) for hematoxylin–eosin staining [41].

Following hematoxylin–eosin staining, the endometria were retrospectively classified into categories I, IIA, IIB, or III based on the Kenney and Doig classification [2]. This classification considers the degree of fibrosis, inflammatory infiltrates, and the extent of dilatation of endometrial glands and lymphatic vessels.

Myometrial samples were retrospectively assigned to categories I, IIA, IIB or III according to the Kenney and Doig classification system [2], in conjunction with an assessment of the phase of the estrous cycle. The same myometria were used for both Experiment 1 and Experiment 2. The present study considered the mid-luteal phase (*n* = 6 category I, *n* = 6 category IIA, *n* = 5 category IIB, *n* = 6 category III) and follicular phase (*n* = 5 category I, *n* = 6 category IIA, *n *= 5 category IIB, *n* = 4 category III) in Experiment 1 and Experiment 2, respectively. The estrous cycle phase was determined by analysing P_4_ levels and observing the ovaries macroscopically [41]. The mid-luteal phase was characterized by a well-developed corpus luteum (CL) associated with follicles of 15–20 mm in diameter and P_4_ levels greater than 6 ng/ml. The follicular phase was characterized by the absence of an active CL, the presence of a follicle larger than 35 mm in diameter and P_4_ levels lower than 1 ng/ml [41].

### Myometrial tissues preparation

In Experiment 1, myometrium was excised from the endometrium and perimetrium of the uterine horns that were ipsilateral to the CL (mid-luteal phase of the estrous cycle) or the growing follicle (follicular phase of the estrous cycle). The myometrium was then washed with cold, sterile, RNAse-free saline solution and placed into RNAlater (Invitrogen, Thermo Fisher Scientific, #AM7021) for determination of *LPA receptor* mRNA transcription using Real-time PCR (qPCR). Before qPCR analysis, myometrial samples were stored at –80 °C until the Kenney and Doig category was assessed [2]. Then, an adequate number of samples were assigned to each group for further analysis.

For experiment 2, myometrial activity was measured by excising a 3–4 mm wide and 6–7 mm long myometrium from the endometrium and perimetrium of uterine horns. The excision was performed ipsilateral to the CL during the mid-luteal phase of the estrous cycle or to the growing follicle during the follicular phase of the estrous cycle. The excised tissue was then cut in the direction of the longitudinal muscle. The myometrium was washed with cold sterile RNAse-free saline solution and placed into 2 ml of aerated physiologic salt solution (PSS; pH 7.4) at 4 °C with 95% air and 5% CO_2_ until measuring myometrial contractility [42]. Measurement of myometrial contractility was performed prior to histopathological classification of the endometrium according to Kenney and Doig [2], with the appropriate number of samples obtained in each experimental group.

## Experimental procedures

### Experiment 1. Myometrial LPA receptors mRNA transcription at different stages of mare endometrosis

To determine the effect of the stage of endometrosis on *LPAR1-LPAR6* mRNA transcription, we used myometrial samples from the mid-luteal phase of the estrous cycle (*n* = 6 category I, *n* = 6 category IIA, *n* = 5 category IIB, *n* = 6 category III) and the follicular phase of the estrous cycle (*n* = 5 category I, *n* = 6 category IIA, *n* = 5 category IIB, *n* = 4 category III). We performed qPCR to measure myometrial mRNA *LPAR1-LPAR6* transcription.

### Experiment 2. The effect of LPA on myometrial contractile activity from different categories of mare's endometrium

To determine the effect of LPA on myometrial contractility at different stages of mare endometrosis, we used myometrial samples from the mid-luteal and the follicular phases of the estrous cycle. The myometrial tissues used in this experiment were from the same uteri as those used in Experiment 1. Myometrial strips were treated with cumulative doses of LPA (10^−8^ M to 10^−6^ M; Sigma Aldrich, #L7260) for 5 min. The concentration of LPA was selected based on a preliminary study (data not shown). The force of myometrial contractions was measured using an isometric contraction transducer.

### Analytic methods

#### Gene expression

Total RNA was extracted from myometrial tissues using the TRI Reagent® (Sigma Aldrich, Germany, #T9424-200 ML) following the manufacturer's instructions. RNA content and purity were evaluated using a NanoDrop 1000 Spectrophotometer (Thermo Fisher Scientific, ND-1000, Wilmington, DE, USA). The A260/280 absorbance ratio for all samples was approximately 2.0, and the 260/230 absorbance ratio ranged between 1.8–2.0. The RNA was reverse transcribed into cDNA using the Reverse Transcription Kit (Qiagen, Hilden, Germany, #205,311) at a concentration of 1.5 μg.

### Real time PCR

Real-time PCR was performed using TaqMan Universal Master Mix II (4,440,049; Applied Biosystems, Foster City, CA, USA) on a Viia7 system (Applied Biosystems, Waltham, Massachusetts, USA) with 384-well plates. All samples were run in duplicate. To measure mRNA transcription of *LPAR1* (cat. no. Ec06980947), *LPAR2* (cat. no. Ec06971360), *LPAR3* (cat. no. Ec07038491), *LPAR4* (cat. no. Ec07035663), *LPAR5* (cat. no. Ec07038512), *LPAR6* (cat. no. Ec04951225), *ubiquitin conjugating enzyme E2B* (UBE2B) (cat. no. Ec07038512), *ribosomal protein L32* (RPL32) (cat. no. Ec06951800_m1), and *succinate dehydrogenase complex subunit A* (SDHA) (cat. no. Ec03470487_m1), Single Tube TaqMan Gene Expression Assays (Life Technologies Thermo Fisher Scientific) were used. The selection of the most suitable reference genes was performed using NormFinder software [43]. To ensure stable expression across the endometrosis categories, gene expression data were normalized to the average geometric mean of the three most stable genes, *SDHA, RPL32,* and* UBE2B.*

### Measurement of myometrial contractility

**Fig. 1:**
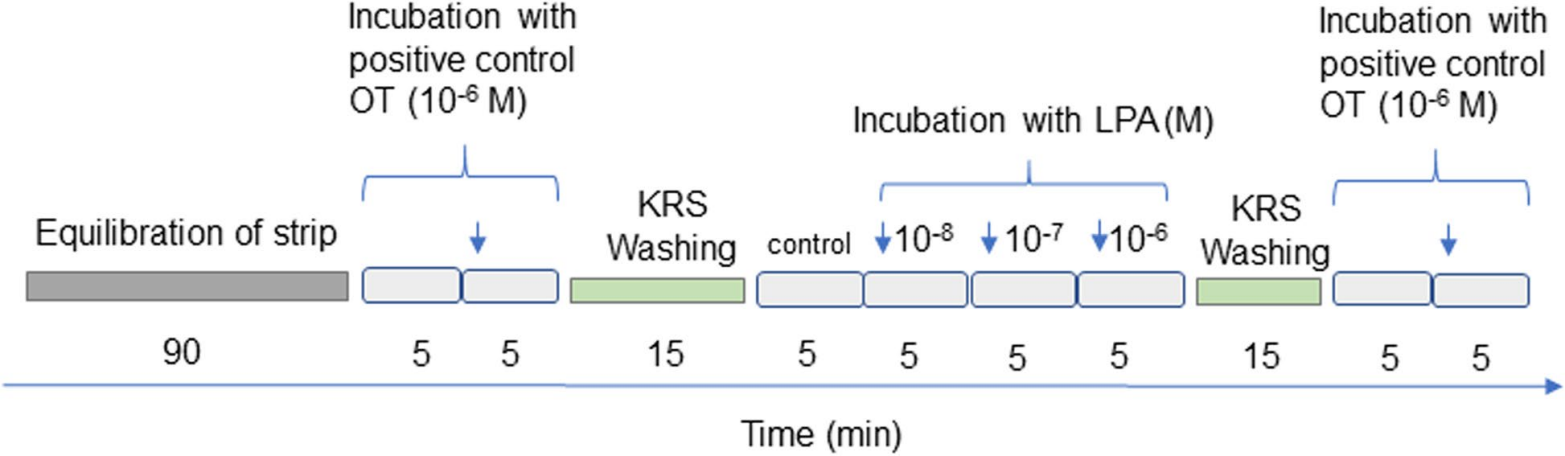
Diagram showing treatment of the myometrial strips. OT – oxytocin; LPA – lysophosphatidic acid; KRS – Krebs–Ringer's solution. Concentrations of the examined substances are expressed in moles (M)

Each myometrial strip was attached individually to the base of the chambers using the HSE Schuler Organ bath apparatus from March-Hugstetten, (Germany). The strip was then tied to the isometric contraction transducer (HSE Type 372) using a stationary hook and surgical silk. Each chamber contained Krebs–Ringer's solution (KRS) with a pH of 7.4 and a volume of 10 ml. The solution was composed of NaCl (120.3 mM), KCl (5.9 mM), CaCl2 (2.5 mM), MgCl2 (1.2 mM), NaH2PO4 (1.2 mM), NaHCO3 (15.5 mM), and glucose (11.5 mM), according to Wrobel et al. [44, 45]. The baths were continuously oxygenated with 95% O_2_ and 5% CO_2_ and maintained at 38.5ºC. The normal rectal temperature of a horse is between 37.7 and 38.9ºC. Therefore, a temperature of 38.5ºC was chosen for the assessment of myometrial contractility, based on our previous experience with in vitro studies using mares` tissue or cells [46]. Figure 1 depicts the experimental protocol for measuring uterine contractility. All preparations were allowed to equilibrate for 90 min. During the pre-incubation period, we observed spontaneous and regular contractions of the myometrial strips (stabilization period). To serve as a positive control, we used oxytocin (OT, 1 µM, Sigma Aldrich, #04375), which clearly stimulated myometrial contraction within 5 min. After 5 min of contraction, the strips were stabilized (data not shown). The dose of OT was chosen based on preliminary studies (data not shown). The study measured the force of isometric contractions of smooth muscle before and after OT application to determine tissue viability and suitability for further study, as previously described [42, 44, 45]. The measurements were taken every 2 s for 5 min during the basal contractions. The chamber was rinsed three times with KRS solution before applying the following factor, in accordance with previous studies [47, 48]. The myometrial contractility was then measured in response to increasing concentrations of LPA (10^−8^ M to 10^−6^ M) for 5 min at each concentration. In the preliminary study (data not shown), the concentrations of LPA were determined. To reassess tissue functionality, OT was administered at the same dose as before. The statistical analysis only considered results in which the difference in response to OT stimulation at the beginning and end of the study was less than 20%.

### Progesterone analysis

Serum P_4_ levels were determined using radioimmunoassay (RIA, Diasource, # KIP1458).

### Statistical analysis

In all experiments, the normal distribution was tested using the Shapiro–Wilk test or D'Agostino & Pearson test. The data were non-parametric. For experiment 1, *LPARs* mRNA transcription in the myometrial tissues was statistically analyzed using two-way ANOVA followed by Sidak's multiple comparison test (GraphPad Software version 8.3.0, GraphPad Software, San Diego, CA, USA). The results were deemed significantly different for values of *P* < 0.05. In experiment 2, we conducted a statistical analysis of the force of myometrial contraction using one-way ANOVA for repeated measures, followed by the Newman-Keuls tests (GraphPad). We expressed the mean (± SEM) values for the contraction force in mN and calculated them using all measurements collected every 2 s for 5 min.

## Results

### Experiment 1. Myometrial mRNA LPA receptors transcription at different stages of mare endometrosis

**Fig. 2:**
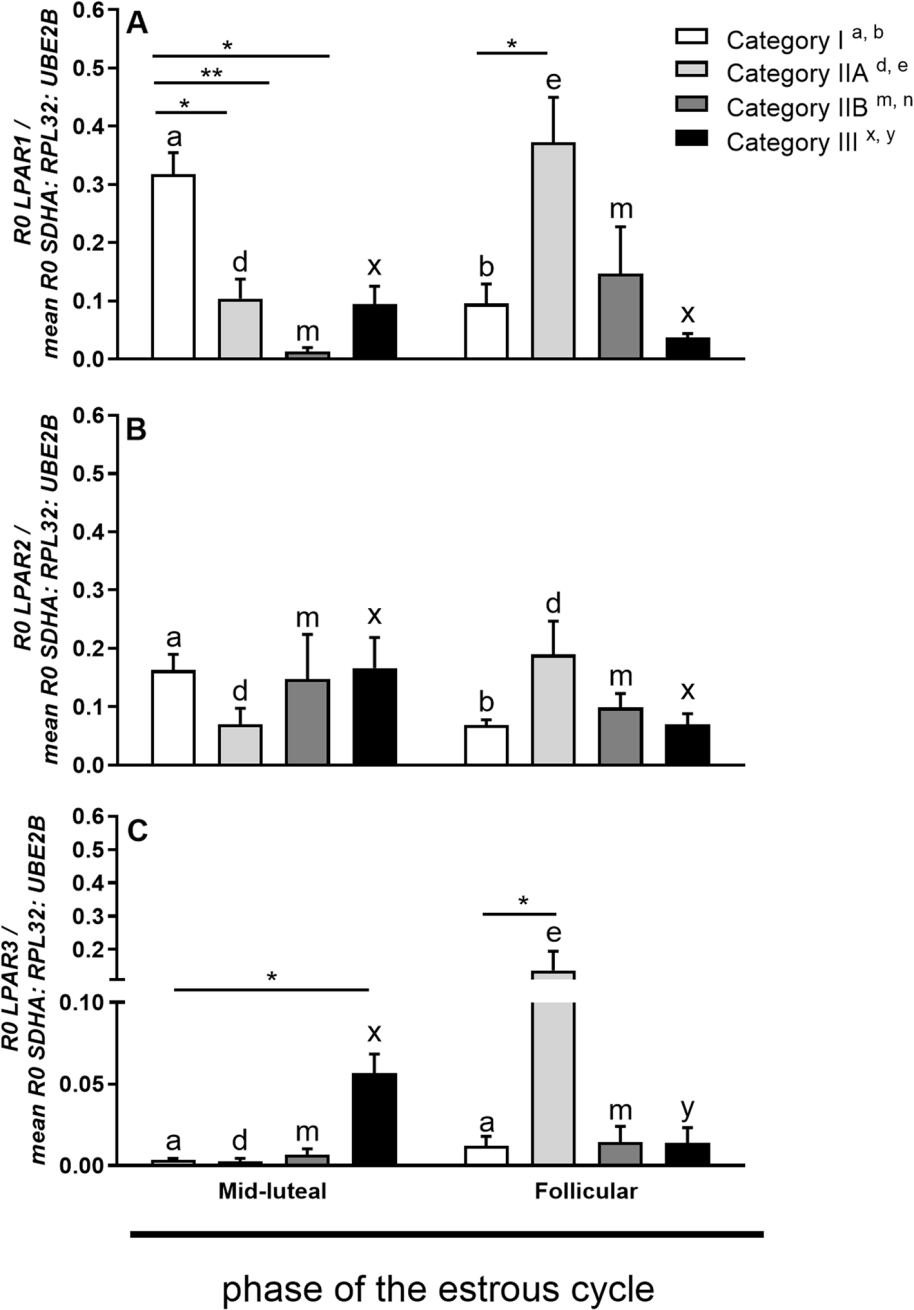
*LPAR1* (**a**), *LPAR2* (**b**), and *LPAR3* (**c**) mRNA transcription during the mid-luteal and follicular phase of the estrous cycle at different stages of endometrosis (Kenney and Doig’s endometrium categories I, IIA, IIB and III). Superscript letters indicate statistical differences between the mid-luteal and follicular phase in Kenney and Doig’s category I ^a,b^ category IIA ^c,d^ category IIB ^e,f^ and category III ^g,h^. Asterisks indicate statistical differences between *LPAR1*, *LPAR2*, *LPAR3* mRNA transcription in endometrosis, within the mid-luteal or follicular phase of the estrous cycle (^*^P < 0.05; ^**^P < 0.01; ^***^P < 0.001)

In mares with category I endometrium, *LPAR1* mRNA transcription was higher in the myometrium during the mid-luteal phase of the estrous cycle compared to its mRNA expression in the myometrium during the follicular phase of the estrous cycle (*P* < 0.05; Fig. 2A). In mares with category IIA endometrium, myometrial *LPAR1* mRNA transcription was lower during the mid-luteal phase of the estrous cycle compared to its expression in the myometrium of mares with category IIA endometrium in the follicular phase of the estrous cycle (*P* < 0.05, Fig. 2A). Furthermore, in the mid-luteal phase of the estrous cycle, *LPAR1* mRNA transcription decreased in the myometrium of mares with endometrium categories IIA, IIB, and III, compared to category I (*P* < 0.05; Fig. 2A). Additionally, during the follicular phase of the estrous cycle, *LPAR1* mRNA transcription was higher in the myometrium of mares with category IIA endometrium than in category I (*P* < 0.05; Fig. 2A).

In mares with endometrium category I, myometrial *LPAR2* mRNA transcription was higher during the mid-luteal phase of the estrous cycle compared to its mRNA transcription in the myometrium of mares with category I endometrium in the follicular phase (*P* < 0.05; Fig. 2B). *LPAR2* mRNA transcription did not differ in the myometrium of mares with endometrium category IIA, IIB, and III between phases of the estrous cycle (*P* > 0.05; Fig. 2B).

In mares with category IIA endometrium, myometrial *LPAR3* mRNA transcription was lower during the mid-luteal phase of the estrous cycle compared to its mRNA transcription in the myometrium of mares with category IIA endometrium in the follicular phase (*P* < 0.05; Fig. 2C). In contrast, the *LPAR3* mRNA transcription of was higher in the myometrium of mares with category III endometrium during the mid-luteal phase of the estrous cycle, compared to its mRNA transcription in the myometrium of mares with category III endometrium in the follicular phase of the estrous cycle (*P* < 0.05; Fig. 2C). During the mid-luteal phase of the estrous cycle, there was a higher *LPAR3* mRNA transcription in the myometrium of mares with category III endometrium compared to category I (*P* < 0.05; Fig. 2C). However, during the follicular phase of the estrous cycle, there was a higher *LPAR3* mRNA transcription in the myometrium of mares with category IIA endometrium compared to category I (*P* < 0.05; Fig. 2C).

In the myometrium of mares with category IIA and III endometria during the mid-luteal phase, and with category IIB and III endometria during the follicular phase, *LPAR4* mRNA transcription was under the level of detection. Therefore, a statistical comparison was not performed for *LPAR4*.

**Fig. 3:**
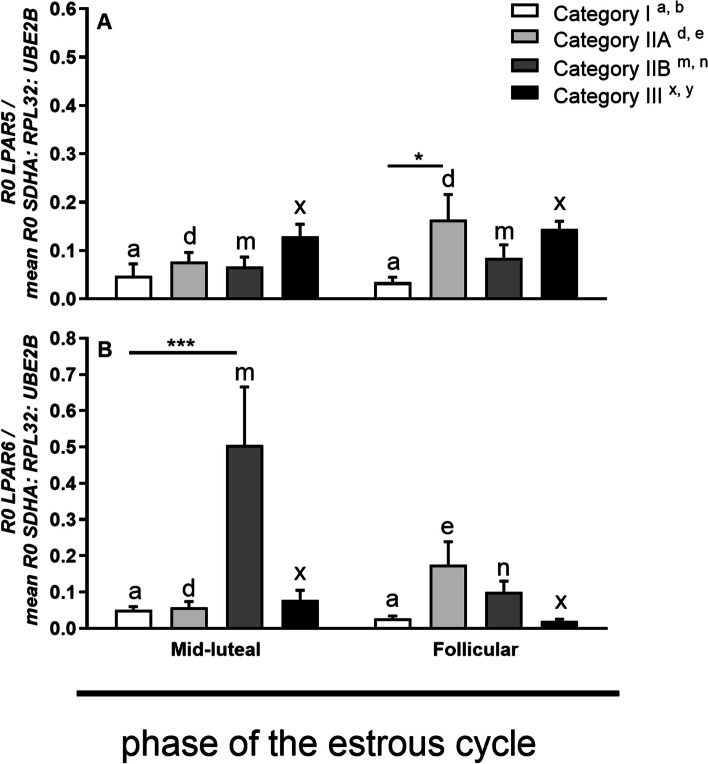
*LPAR5* (**a**), and *LPAR6* (**b**) mRNA transcription during the mid-luteal and follicular phase of the estrous cycle at different stages of endometrosis (Kenney and Doig’s endometrium categories I, IIA, IIB and III). Superscript letters indicate statistical differences between the mid-luteal and follicular phase in Kenney and Doig’s category I ^a,b^ category IIA ^c,d^ category IIB ^e,f^ and category III ^g,h^. Asterisks indicate statistical differences between *LPAR5*, *LPAR6* mRNA transcription in endometrosis, within the mid-luteal or follicular phase of the estrous cycle (^*^P < 0.05; ^**^P < 0.01; ^***^P < 0.001)

*LPAR5* mRNA transcription did not differ in the myometrium of mares with category I, IIA, IIB, and III endometria between phases of the estrous cycle (*P* > 0.05; Fig. 3A). During the follicular phase of the estrous cycle, *LPAR5* mRNA transcription was higher in the myometrium of mares with category IIA endometrium, compared with category I (*P* < 0.05; Fig. 3A).

*LPAR6*mRNA transcription was lower in the myometrium of mares with category IIA endometrium in the mid-luteal phase compared to its mRNA transcription in the myometrium of mares with category IIA endometrium in the follicular phase of the estrous cycle (*P* < 0.05; Fig. 3B). While, in mares with category IIB endometrium, *LPAR6* mRNA transcription was higher in the mid-luteal phase compared to its mRNA expression in the myometrium of mares with category IIB endometrium in the follicular phase (*P* < 0.001; Fig. 3B). Additionally, *LPAR6* mRNA transcription was higher in the myometrium of mares with category IIB endometrium compared to category I in the mid-luteal phase (P < 0.001; Fig. 3B).

### Experiment 2. The effect of LPA on myometrial contractile activity from different categories of mare's endometrium

**Fig. 4:**
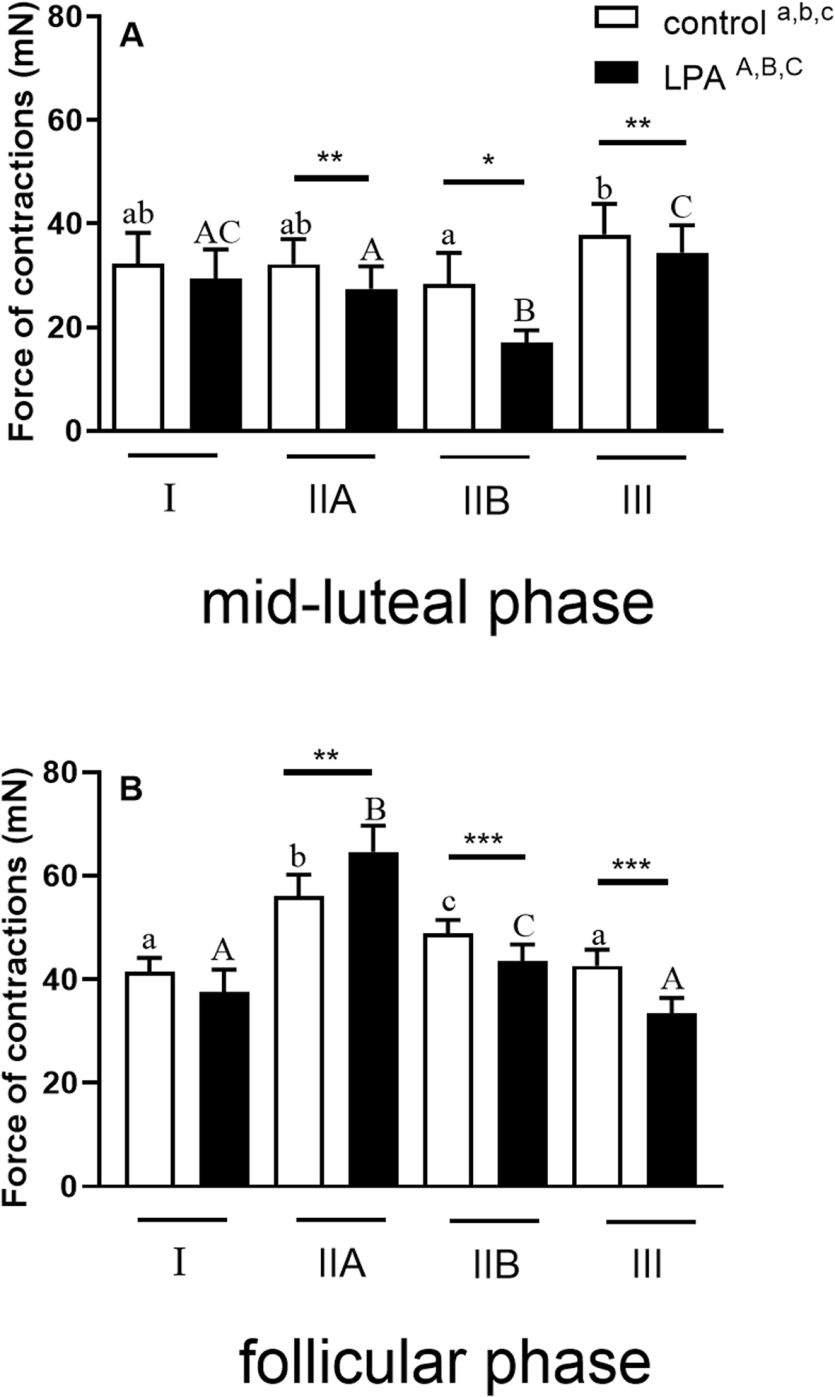
The mean (± SEM) basal (white bars) and LPA -stimulated (cumulative dose 10-6 M; bars with patterns) force of the contractions of the myometrial strips during: (**a**) mid-luteal, or (**b**) follicular phase of the estrous cycle at different stages of endometrosis (Kenney and Doig’s endometrium categories I, IIA and IIB and III). Asterisks indicate statistical differences in the force of the contractions between control and LPA-stimulated myometrial strip within each stage of endometrosis (*P < 0.05; **P < 0.01; ***P < 0.01). Different superscript letters indicate statistical significance (*P* < 0.05) within control groups (control ^a,b,c^) or within LPA-stimulated myometrial strips between each stage of endometrosis (LPA^A, B,C^)

The objective of this experiment was to compare the force of myometrial contractions in mares during the mid-luteal phase of the estrous cycle between control and LPA-treated groups at different stages of endometrosis. The cumulative dose of LPA (10^−8^ M to10^−6^ M) decreased the myometrial contraction force in mares with categories IIA (*P* < 0.01), IIB (*P* < 0.05), and III (*P* < 0.01) endometria compared to their respective control groups: control IIA, control IIB, and control III (Fig. 4A). Furthermore, a comparison of myometrial contraction force was conducted within the control groups during the mare endometrosis. A reduction in the force of myometrial contraction was observed in the control group IIB in comparison to the control group III (*P* < 0.001; Fig. 4A). A comparison of myometrial contraction force was compared within LPA-treated groups revealed a decrease in mares with category IIB endometria compared to mares with categories I, IIA, and III endometria (*P* < 0.001; Fig. 4A). However, the myometrial contraction force in mares with category III endometrium was observed to be higher than that observed in mares with categories IIA and IIB endometria (*P* < 0.05 and *P* < 0.001; respectively; Fig. 4A).

Furthermore, the force of myometrial contractions in mares during the follicular phase of the estrous cycle was compared between control and LPA-treated groups at different stages of endometrosis. The cumulative dose of LPA (ranging from 10^−8^ M to 10^−6^ M) was found to increase the force of myometrial contractions in mares with category IIA endometrium, as compared to the control group IIA (*P* < 0.01). However, LPA was observed to decrease the force of contractions in mares with categories IIB (*P* < 0.001) and III endometria (*P* < 0.001), in comparison to their respective control groups: group IIB, and group III (Fig. 4B). Furthermore, the force of myometrial contractions was compared within control groups during the mare endometrosis. An increase in the force of myometrial contraction was observed in the control group IIA and control group IIB, as compared to the control group I and control group III; respectively ( *P* < 0.01; Fig. 4B). Furthermore, the force of myometrial contraction was observed to be higher in the control group IIA in comparison to the control group IIB (*P* < 0.001; Fig. 4B). A comparison of myometrial contraction force within LPA-treated groups revealed an increase in mares with category IIA and IIB endometria compared to category I and III endometria, respectively (*P* < 0.01; Fig. 4B). However, the force of myometrial contraction was higher in mares with category IIA endometria compared to mares with category IIB endometria (*P* < 0.001; Fig. 4B).

## Discussion

To the best of our knowledge, this is the first pilot study to demonstrate the mRNA transcription of *LPARs* in the myometrium of mares with endometrosis during both the mid-luteal and follicular phases of the estrous cycle. In addition, our study is the first to investigate whether myometrial contractility in response to LPA varies according on the stage of endometrosis in mares.

Lysophosphatidic acid is mediated by six receptors in the organism [49]. High mRNA transcription levels of *LPAR1* and *LPAR3* have been reported in reproductive organs, such as the ovary and uterus, during the estrous cycle and early pregnancy in different species [23–31, 50–52], indicating the importance of LPA-mediated signaling in the reproductive processes. In our study, *LPARs* are expressed at different mRNA levels in the equine myometrium during endometrosis. *LPAR1* mRNA was reduced in mares with endometrosis during the mid-luteal phase, whereas *LPAR3* was upregulated in category III. During the follicular phase, *LPAR1*, *LPAR3*, and *LPAR5* were upregulated in category IIA compared to category I.

Szóstek-Mioduchowska et al. [40] recently reported changes in the endometrial concentration of LPA, alterations in *LPAR* mRNA transcription and protein abundance at different stages of endometrosis in mares. We found higher myometrial mRNA transcription of *LPAR6* in category IIB compared to category I during the mid-luteal phase. Yukiura et al. [53] reported high *LPAR6* mRNA expression in human umbilical vein endothelial cells, which regulate blood vessel formation. While in buffalo, the upregulation of *LPAR6* mRNA in the endometrium suggests a role for LPAR6-mediated signaling in early pregnancy [52]. Further studies are needed to explore LPAR6-mediated pathways in equine uterine vascular regulation during endometrosis.

Yung et al. [20] suggest that LPAR2, LPAR4, and LPAR5 have little impact on reproduction. Our study shows that myometrial mRNA transcription of *LPAR4* mRNA transcription was not detected in most of the myometrium examined. Nevertheless, it is proposed that signaling by *LPAR2*, *LPAR4*, and *LPAR5* may have a limited effect on myometrial contractility in mares.

We assumed that the myometrium contractility could be affected by changes in *LPAR* mRNA transcription. However, we observed variability in *LPAR* mRNA transcription at different phases of the estrous cycle and at different stages of endometrosis. Unfortunately, a posttranscriptional analysis of LPARs was not performed in the presented study. The mRNA levels do not always correlate directly with protein expression. Therefore, it can be postulated that discrepancies between the transcription level and the myometrial contraction patterns may be explained by different posttranscriptional changes in LPAR expression within the myometrium during the progression of endometrosis. It is important to note that ovarian steroid hormones have previously been demonstrated to regulate LPAR mRNA and protein levels in mice [54, 55] and sheep [31] uteri. Moreover, Boruszewska et al. [29] demonstrated that LPA has a stimulatory effect on E_2_ synthesis, which is likely mediated by increased expression of the FSHR and 17β-HSD genes in bovine granulosa cells. Therefore, the role of ovarian steroids in potentially influencing *LPAR* expression in our study, which may contribute to the variability in *LPAR* transcription observed in different phases of the estrous cycle and stages of endometrosis should also be considered. In future studies, it would be beneficial to obtain a more comprehensive hormonal profile of blood samples. Therefore, in addition to measuring progesterone, estradiol concentrations should also be assessed in the mares from which uteri are collected for the in vitro studies.

Previous studies have shown that LPA plays a crucial role in the regulation of myometrial contractility in rats [22, 23] and pigs [34]. In addition, LPA stimulated smooth muscle contraction and intrauterine pressure in rats in cooperation with uterotonic prostaglandin F_2α_ (PGF_2α_) [22, 23]. However, the role of LPA in equine myometrial function is not fully understood. The function of the equine myometrium is essential from a clinical perspective. The uterus is cleaned by contractile activity and immune cells that naturally infiltrate the uterus. Therefore, a reduction in myometrial activity can lead to problems with uterine cleansing, resulting in persistent inflammation in mares, known as endometritis. Several reports suggest that this inflammation may be associated with the development of endometrosis in mares [56–58]. Mares with endometrosis often experience prolonged endometritis, which can lead to the development of fibrosis due to the presence of active immune cells in the uterus. This in turn, leads to an overproduction of inflammatory mediators [59, 60]. Therefore, the proper function of the myometrium is linked to the network of pathways that regulate contractility and inflammatory responses in the equine myometrium.

In the preliminary study, OT, the positive control, was shown to increase myometrial contractility. This result is consistent with other in vitro myometrial studies in mares, where OT was required to stimulate initial contractile patterns in the muscle layers prior to analysis [61–63]. It is important to note that uterine contractility is essential for sperm transport and is a critical component in the clearance of acute inflammatory debris as a result of bacterial and sperm entry into the mare`s uterus [38]. As the myometrium is composed of a strong inner circular layer and a thin outer longitudinal smooth muscle layer, the circular muscles may play a more active role in myometrial contractions during estrus than the longitudinal muscles. However, the longitudinal muscles are physiologically more active than the circular muscles during the luteal phase of the estrous cycle [38]. Therefore, the longitudinal muscles were chosen for our pilot studies.

The effect of LPA dependents on the species and the physiological status of the animal. Markiewicz et al. [34] reported that increasing doses of LPA (10^−7^ M, 10^−6^ M, 10^−5^ M) caused an increase in contraction tension, amplitude, and frequency of strips consisting of endometrium with myometrium in the uterine horn with developing embryos in pigs. This effect was not observed in myometrial strips from the uterus without embryos [34]. Hama et al. [64] showed that LPA induces smooth muscles contraction in the pregnant mouse uterus, and plays a novel role in labor and parturition in rats [33]. In addition, LPA (10^−6^ M) increased stress fibers formation in cultured human myometrial cells. This has been shown to contribute to smooth muscle contraction [32]. However, Toews et al. [65] investigated the effect of LPA on the contractile responsiveness of isolated rabbits and cats tracheal rings and found that LPA alone did not induce contraction on its own in either species. Interestingly, our study found no effect of LPA on myometrial contractility in mares with category I endometrium in both the mid-luteal and follicular phases of the estrous cycle. We hypothesize that this lack of effect may be due to LPA affecting other pathways or processes in the mare’s uterus that are unrelated to myometrial contraction. Lysophosphatidic acid is known to activate several signaling cascades, some of which mediate smooth muscle contraction, while others are involved in physiological or pathological processes [19, 20]. Lysophosphatidic acid receptors are involved in processes such as cell proliferation, migration, cytoskeletal reorganization [20, 66, 67], cytokine/chemokine production [68], platelet aggregation [69], tissue inflammation or remodeling [70], and others. On the other hand, LPA receptors play a role in pathological conditions such as fibrosis, reproductive disorders, and bone metabolism, and cancer [67, 71] The results of our study showed that the effect of LPA on uterine contractility depends on the histological grade of the endometrium. We showed that in mares with endometrosis, LPA decreased myometrial contractile activity in the mid-luteal phase, whereas in the follicular phase, myometrial contractility decreased in categories IIB and III and increased in category IIA. According to Szóstek-Mioduchowska et al. [40], LPA may indirectly promote fibrogenesis by acting on PGF_2α_ in the equine endometrium during endometrosis. In addition, the mare's myometrium has been shown to produce PG [72]. Therefore, it is possible that LPA indirectly stimulates fibrogenesis and mediates myometrial contractility by affecting PG secretion in the mare's myometrium, leading to dysfunction during the estrous cycle. This may also explain the inconsistent pattern of changes observed in myometrial contractility in response to LPA. Further studies are needed to investigate whether the response of the myometrium to LPA is different in mares with endometriosis, when PG secretion in the endometrium is disturbed.

Dancs et al. [73] reported that LPAR1-mediated thromboxane A2 release is responsible for LPA-induced vascular smooth muscle contraction in mice, indicating that LPAR1 contributes to vasoregulation and remodeling. In addition, it should be considered that vascular elastosis could affect blood flow to the myometrium, potentially impairing uterine contractility. Elastosis is characterized by thickening of the elastin layers within the uterine vasculature, resulting in decreased blood flow and uterine perfusion [11, 74, 75]. Vascular elastosis has been reported in aged, multiparous, and infertile mares, and in mares with chronic uterine endometritis. Therefore, further research is needed to investigate the extent of these vascular changes and their impact on myometrial function in the progression of endometrosis in mares.

The results of our pilot study are promising. However, there are some limitations to our study. Myometrial contraction is regulated by several factors, including the autonomic nervous system, endocrine, paracrine, and autocrine factors. Disruptions in the expression of any of these regulatory factors could potentially impact the expression of other molecules involved in maintaining endometrial and myometrial homeostasis and related physiological processes. It is hypothesized that LPA may modulate a step in the myometrial contractile signaling pathway that is shared by other factors/agents, rather than acting solely on the receptor. Endometrosis is known to be a complex disease, associated with chronic infection. Mares with endometrosis show uterine smooth muscle atrophy, myometrial collagen fiber hyperplasia [11], impaired lymphatic drainage, fluid retention, and myometrial vascular degeneration [12]. Therefore, several factors should be considered to fully understand its progression and impact on myometrial tissue. Equine endometrosis is more commonly observed in older animals, as it tends to develop with the age of the mare. The myometrial alterations observed in this study can reasonably be attributed to several factors, including the age of the mares, their history of recurrent or chronic inflammatory processes often associated with advanced age or uterine pathology, as well as the number of foals produced and the mares' breeding status. [1, 76]. The age range of the mares used in our study was between 2 and 18 years. The wide age range of the animals was chosen to provide a cross-sectional view of endometrial fibrosis at different stages of its progression. As noted by Ebert et al. [77], endometrosis tends to increase with age, affecting 32% of mares under 5 years of age and 93% of mares over 20 years of age. By including mares across this wide age range, our study aimed to capture different stages of the disease. As we found that the effects of LPA on myometrial contractility varied between different categories and estrous cycle phases, individual differences between the mares may have influenced the results. Therefore, the number of animals used in the study may have contributed to the observed variability in the results, as well as the lack of detailed reproductive histories of the individual mares.

To better understand the relationship between LPA and endometrosis, further research involving immunohistochemical analysis or protein expression of LPARs in myometrial tissue during the endometrosis progression is needed. As mentioned above, Szóstek-Mioduchowska et al. [40] highlighted different patterns of LPAR expression at both mRNA and protein levels. It is important to note that mRNA levels do not always correlate directly with protein expression or localization. Therefore, discrepancies between *LPAR* mRNA expression and the observed changes in myometrial contractility in response to LPA could be due to differences in receptor expression at the gene and protein level. Given the pivotal nature of this study, we plan to address this limitation in future work. In addition, potential interactions between the endometrium and myometrial tissue during the progression of endometrosis in mares should be investigated in future studies.

In summary, altered patterns of *LPAR* mRNA transcription were observed in the myometrium of mares during the progression of endometrosis. On the one hand, we found a decrease in myometrial *LPAR1* mRNA transcription in the mid-luteal phase during the progression of endometrosis, while, upregulation of myometrial *LPAR3* or *LPAR6* mRNA transcription in mares with category III or IIB endometria. On the other hand, an increase in myometrial *LPAR1*, *LPAR3*, *LPAR5* mRNA transcription was observed in the follicular phase in mares with category IIA endometria. However, LPA had no significant effect on myometrial contractions in mares with category I endometrium during both phases of the estrous cycle. This lack of the effect may be due to LPA affecting other pathways or processes in the mare’s uterus that are unrelated to myometrial contractions. However, as endometrosis progresses, the effect of LPA on myometrial contractility appears to vary depending on the category of endometrium and the phase of the estrous cycle. Due to possible interactions between LPA and other signalling pathways in the mare`s uterus, the role of LPA in myometrial contractility remains unclear. Therefore, further studies are needed to investigate the effect of LPA on other LPA-regulated signalling pathways in the mare`s myometrium.

## Conclusion

In the course of endometrosis in mares, a disruption in the myometrial mRNA transcription of *LPARs* has been observed. This is the first study to examine the impact of LPA on myometrial contractility at different stage of endometrosis. However, it is essential to consider that multiple factors may contribute to this process. Alternations in contractile activity and changes in myometrial *LPARs* mRNA transcription may indicate impaired LPA-signaling mechanisms in equine myometrium during endometrosis.

## Data Availability

The datasets used and analysed during the current study are available from the corresponding author on reasonable request.

## Abbreviations

CL: Corpus luteum
GPCRs: G-protein-coupled receptors
KRS: Krebs-Ringer's solution
LPA: Lysophosphatidic acid
LPARs: LPA receptors
OT: Oxytocin
P_4_: Progesterone
PG: Prostaglandin
PGF_2α_: Prostaglandin F_2α_
PSS: Physiologic salt solution
qPCR: Real-time PCR
RPL32: *Ribosomal protein L32*
SDHA: *Succinate dehydrogenase complex subunit A*
UBE2B: *Ubiquitin conjugating enzyme E2 B*
